# EGFR and PI3K Pathway Activities Might Guide Drug Repurposing in HPV-Negative Head and Neck Cancers

**DOI:** 10.3389/fonc.2021.678966

**Published:** 2021-06-11

**Authors:** Andreas Mock, Michaela Plath, Julius Moratin, Maria Johanna Tapken, Dirk Jäger, Jürgen Krauss, Stefan Fröhling, Jochen Hess, Karim Zaoui

**Affiliations:** ^1^ Department of Medical Oncology, National Center for Tumor Diseases (NCT) Heidelberg, Heidelberg University Hospital, Heidelberg, Germany; ^2^ Division of Translational Medical Oncology, NCT Heidelberg, German Cancer Center (DKFZ), Heidelberg, Germany; ^3^ Department of Otorhinolaryngology, Head and Neck Surgery, Heidelberg University Hospital, Heidelberg, Germany; ^4^ Department of Oral and Cranio-Maxillofacial Surgery, Heidelberg University Hospital, Heidelberg, Germany; ^5^ Molecular Mechanisms of Head and Neck Tumors, DKFZ, Heidelberg, Germany

**Keywords:** precision oncology, targeted therapy, head and neck squamous cell carcinoma, omics, systems biology

## Abstract

While genetic alterations in Epidermal growth factor receptor (EGFR) and PI3K are common in head and neck squamous cell carcinomas (HNSCC), their impact on oncogenic signaling and cancer drug sensitivities remains elusive. To determine their consequences on the transcriptional network, pathway activities of EGFR, PI3K, and 12 additional oncogenic pathways were inferred in 498 HNSCC samples of *The Cancer Genome Atlas* using PROGENy. More than half of HPV-negative HNSCC showed a pathway activation in EGFR or PI3K. An amplification in EGFR and a mutation in PI3KCA resulted in a significantly higher activity of the respective pathway (p = 0.017 and p = 0.007). Interestingly, both pathway activations could only be explained by genetic alterations in less than 25% of cases indicating additional molecular events involved in the downstream signaling. Suitable *in vitro* pathway models could be identified in a published drug screen of 45 HPV-negative HNSCC cell lines. An active EGFR pathway was predictive for the response to the PI3K inhibitor buparlisib (p = 6.36E-03) and an inactive EGFR and PI3K pathway was associated with efficacy of the B-cell lymphoma (BCL) inhibitor navitoclax (p = 9.26E-03). In addition, an inactive PI3K pathway correlated with a response to multiple Histone deacetylase inhibitor (HDAC) inhibitors. These findings require validation in preclinical models and clinical studies.

**Graphical Abstract:**
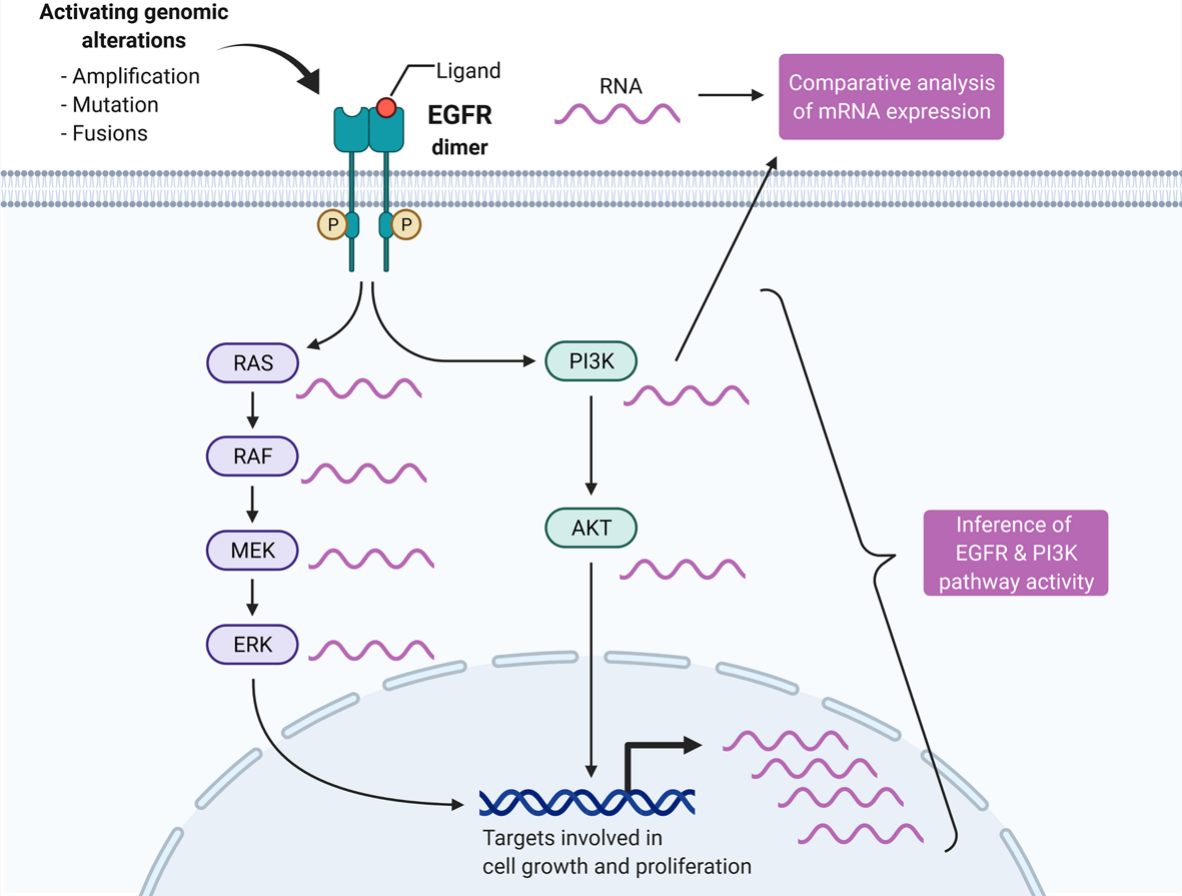


## Introduction

Head and neck squamous cell carcinoma (HNSCC) is the sixth most common cancer worldwide with an incidence that is predicted to increase by 30% by 2030 (1). Risk factors include smoking, alcohol abuse, and for oropharyngeal cancers infection with the human papillomavirus (HPV). The treatment approach is guided by the anatomical site, stage, and pathological risk factors such as the nodal or HPV status. Locally advanced HNSCC is treated in a curative intent with a multimodal approach including surgery, radiotherapy, and chemotherapy. Recurrent and/or metastatic (R/M) disease is treated with palliative systemic therapies in case a salvage resection, re-irradition (especially nasopharyngeal cancers) (2), or metastasectomy (especially HPV+ tumors) (3) is not feasible.

First-line palliative treatment includes the immune checkpoint inhibitor pembrolizumab in patients with PDL1-expressing (CPS>1) tumors or tumors with microsatellite instability in the absence of a contraindication to immunotherapy. A combination of pembrolizumab with chemotherapy should be considered for patients with a high tumor burden or a CPS <20 (4). For PDL1-negative tumors, the treatment standard remains the EGFR targeting antibody cetuximab in combination with a platinum compound and 5FU. While the molecular landscape of HNSCC has been known for years (5), cetuximab remains the only approved targeted therapy in HNSCC.

Beyond EGFR targeting, the PI3K/AKT/mTOR pathway has been extensively studied in HNSCC. Genetic alterations in the pathway are among the most frequent (13–56%) in HNSCC and are independent of the HPV status (5). mTOR inhibitors were the first to be tested in HNSCC as a monotherapy [everolimus (6), temsirolimus (7)] and in a combination with erlotinib (8, 9), but did not show a clinical benefit in terms of median progression free survival (PFS) or overall survival (OS). Also, the pan-PI3K inhibitor PX-866 did not improve PFS, overall response rate (ORR), or OS in pretreated R/M HNSCC when combined with docetaxel or cetuximab (10, 11). Buparlisib, a selective PI3K inhibitor, has been the only drug targeting the PI3K/AKT/mTOR pathway to show PFS and survival benefit in a randomized phase II trial in combination with paclitaxel *vs.* placebo and paclitaxel (12). Of note, this benefit came at the price of high toxicity while the cohort was not stratified for genetic alterations in the PI3K/AKT/mTOR pathway. Hence, reliable biomarkers are needed to predict the response to buparlisib.

In the search for novel predictive molecular biomarkers, most precision oncology programs and umbrella biomarker-driven trials rely on genetic alterations in tumors determined by panel, exome, or whole-genome sequencing (13–18). Common to these studies is a clinical benefit rate associated with molecularly informed therapy decisions of about one-third across tumor types. To increase this percentage, transcriptomics has been considered the next frontier in precision cancer medicine (19). On one hand, it has been showed that transcriptomics increases the number of targetable molecular changes compared to genomics profiling alone (20). On the other hand, gene expression was found to have more power to predict cancer cell vulnerabilities *in vitro* than genomics (21). However, the development of robust expression-based biomarkers is considerably more challenging than DNA-based biomarkers due to the larger scale of features, reproducibility, and variability between assays. A very promising strategy to circumvent these issues are pathway-level biomarkers (22, 23).

In this study, pathway activity inference was performed on bulk transcriptomes of HNSCCs to investigate the impact of genetic alterations on EGFR and PI3K expression and on the respective pathway activation. Suitable *in vitro* pathway models could be identified for both EGFR and PI3K pathway activation. Matching drug screen data enabled a drug sensitivity analysis. The findings of this study could help to guide drug repurposing and the clinical trial design in HNSCC.

## Materials and Methods

### Expression and Clinical Data

RNA-seq data of the TCGA-HNSC cohort was retrieved *via* recount2 (24). The RangedSummarizedExperiment object contained the counts summarized at the gene level using the Gencode v25 (GRCh38.p7, CHR) annotation. TPM (transcripts per million) values were calculated from the count data to normalize for library size and gene length. Matching clinical and genetic data were available for a total of 498 samples and obtained through cBioportal (https://www.cbioportal.org/, accessed on 2020-09-07). The TCGA-HNSC cohort comprised both HPV-positive (n = 413) and HPV-negative tumors (n = 70). **Table S1** lists the TCGA barcodes of the 498 samples included in this analysis.

The microarray data of the HIPO-HNSC (Heidelberg Institute for Personalized Oncology) cohort (n = 79) used in this study is deposited at the Gene Expression Omnibus under the accession number GSE117973. For a detailed description of the clinicopathological characteristics of the cohort, please refer to Schmitt et al. (25). The raw intensity files of the HumanHT-12 BeadChip array (Illumina) were qspline normalized (affy R package) and median centered.

Gene expression microarray data of 45 HPV-negative head and neck carcinoma cell lines from Lepikhova et al. (26) were available under the GEO accession number GSE108062. The lumi R package was used for quantile normalization to the make expression values comparable across microarrays (27).

The gene-level normalized RNA-seq data (log2 transformed tpm values using a pseudo-count of 1; version 20Q3) of 26 HNSCC cell lines of the Cancer Cell Line Encyclopedia (CCLE) project were obtained through the Dependency Map (DepMap) portal of the Broad Institute (28). The DepMap identifiers of the CCLE cell lines used are listed in **Table S2**.

### Drug Sensitivity Data

The drug sensitivity data of 45 HPV-negative head and neck carcinoma cell lines were obtained from the supplemental material of Lepikhova et al. (26). The authors screened in total 220 drugs including both FDA-approved investigational agents and quantified the compound response by a model-based drug-sensitivity score (DSS). A high DSS corresponded to a higher sensitivity, i.e., lower concentrations needed to decrease cell viability. For a detailed description of the testing and scoring, please refer to Lepikhova et al. (26).

The drug sensitivity data of the validation cohort of 26 HNSCC cell lines of the PRISM project (Version 19Q4) were downloaded from the DepMap portal of the Broad Institute (29).

### Pathway Activity Inference

Pathway activities were inferred using the PROGENy algorithm implemented in the *progeny* R package (22). PROGENy leverages a large collection of publicly available perturbation experiments that were used to create a list of pathway response genes that are the basis for the inference. Version 1.10 of the R package enables inference of 14 pathways involved in tumorigenesis (Androgen, EGFR, Estrogen, Hypoxia, JAK-STAT, MAPK, NFkB, p53, PI3K, TGFb, TNFa, Trail, VEGF, WNT). Input to PROGENy were the normalized RNA-seq (tpm values) and microarray data (quantile normalized), respectively. To enable a comparison between the analyzed tumor and cell line samples, the pathway activities were scaled within the individual cohort (separate scaling for TCGA, HIPO and cell line cohort).

### Statistical Analysis

The statistical analysis was conducted in R (version 4.0.2). Heatmaps were generated with the *ComplexHeatmap* R package. The *survminer* R package was used for plotting Kaplan–Meier curves. In the survival time analysis, p-values were calculated by log-rank test. Multiplicity unadjusted P values are presented. In the drug sensitivity analysis, the impact of the EGFR and PI3K pathway activity was modeled as a continuous parameter in a univariate linear regression model. The filters for the selection of top candidate drugs were a p-value cutoff of 0.01, an absolute coefficient of the linear model of 2 and a per-drug median sensitivity score of 2.168, which corresponded to the median of the distribution of all measured drug sensitivity scores in the experiment of Lepikhova and colleagues (26).

## Results

### EGFR and PI3K Pathway Activity Inference and Genetic Alterations in HNSCC

**Figure 1:**
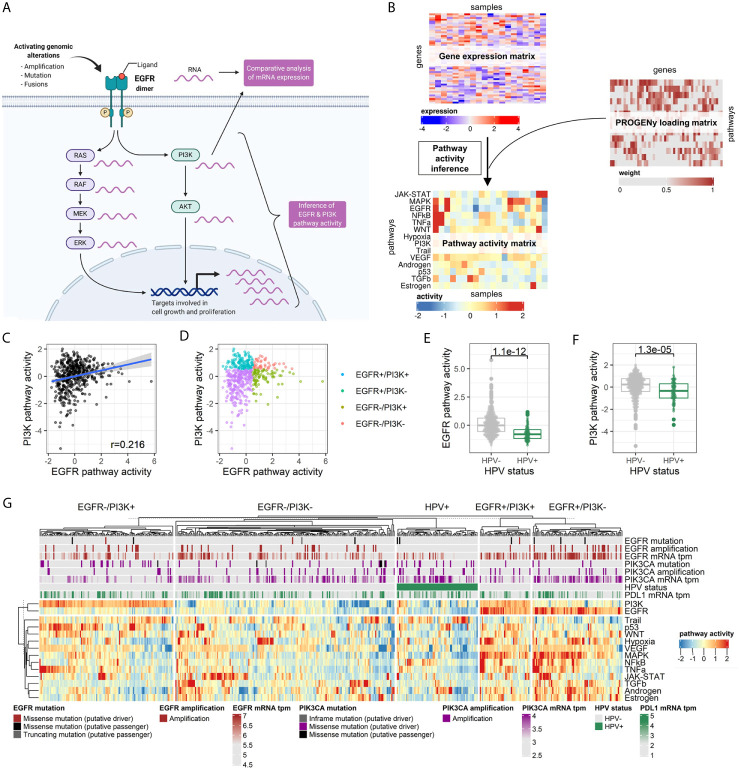
Pathway activity inference in head and neck cancer tissues. **(A)** Graphical abstract of the approach. In contrast to differential mRNA expression analysis of genes of interest (e.g., EGFR and PI3KCA), pathway activity can be statistically inferred by integrating expression values of all genes known to be perturbed upon pathway alteration. Adapted from “HER2 Signaling Pathway”, by BioRender.com (2020). Retrieved from https://app.biorender.com/biorender-templates. **(B)** The PROGENy algorithm is used to infer the activity of 14 key pathways involved in oncogenesis. Activities are calculated by matrix multiplication of the normalized gene expression matrix and the so-called PROGENy loading matrix that contains the full human pathway model of 22,479 genes with associated pathways, weights, and p-values (22). **(C)** Correlation between EGFR and PI3K pathway activity across the cohort. **(D)** Pathway activity-based grouping of HNSCC tumors. **(E)** EGFR and **(F)** PI3K pathway activity stratified by HPV status. **(G)** Heatmap of pathway activity matrix of the TCGA-HNSC cohort (n = 498). The column annotation contains genetic alterations and normalized mRNA expression values of genes of interest, as well as the HPV status.

The activity of EGFR, PI3K, and 12 other key oncogenic pathways was inferred by the PROGENy algorithm from the normalized RNA-seq data of the TCGA-HNSC cohort consisting of 498 tumor samples (**Figures 1A, B**). The pathway activity score was scaled relative to the whole cohort, and a score of 0.5 corresponding to the top quartile of scores was considered an activation. The activity scores of EGFR were not significantly correlated with PI3K (r = 0.216, Pearson correlation; **Figure 1C**), but with MAPK (r = 0.794, Pearson correlation; **Figure S1**). The 498 tumor samples were grouped according to EGFR and PI3K pathway activation (**Figure 1D**). Human papillomavirus positive samples (HPV+) were grouped separately as they were found to harbor significantly lower EGFR and PI3K pathway activities (p = 1.10E-12 and p = 1.30E-05, respectively, Student’s t-test; **Figures 1E, F**). Likewise, HPV+ cases were significantly enriched in the EGFR-/PI3K- group (p = 5.65E-08, Fisher’s Exact Test; **Figure S2**). The most prevalent subgroup in the TCGA-HNSC cohort were EGFR-/PI3K- tumors (38%), followed by EGFR-/PI3K+ (23%), EGFR+/PI3K- (16%), HPV+ (14%), and EGFR+/PI3K+ tumors (9%; **Figure 1G**). Consequently, more than half (56%) of HPV-negative (HPV-) tumors showed a pathway activation in at least one of the two pathways. EGFR was mutated in 13 tumors (3%), but only three mutations were classified as putative drivers. EGFR amplification was observed in 53 cases (11%), which were mutually exclusive with putative driver mutations. PI3KCA mutations were identified in 55 cases (11%) with 50 cases being classified as putative driver mutations. Amplifications in PI3KCA were found in another 11% of cases (n = 57). One-fourth of all tumors in the cohort had a genetic alteration in PI3KCA. As PDL1 protein expression is an established predictive biomarker in HNSCC, we performed a comparative analysis of mRNA expression. No difference in PDL1 mRNA expression could be found between the pathway activity-defined groups (**Figure S3**). In a survival time analysis with overall survival as the endpoint, no prognostic difference could be observed between the four HPV- groups (p=0.670). In line with published data, the HPV- tumors had a more dismal prognosis in contrast to HPV+ tumors (p = 0.006, **Figure S4**). To validate the presence of these four groups of HPV- HNSCC, we performed PROGENy on a second independent HNSCC cohort of the Heidelberg Institute of Personalized Oncology (HIPO, n = 79). While there was a higher fraction of HPV+ tumors in this cohort (29%), all four HPV- pathway activity groups could be identified (**Figure S5**).

### Impact of Genetic Alterations on EGFR and PI3K Pathway Activity and Gene Expression

**Figure 2:**
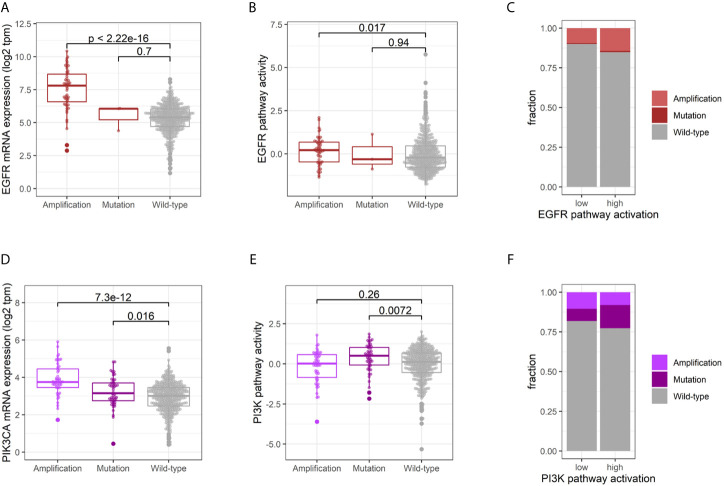
Impact of genetic alterations on EGFR and PI3K activity. Comparative EGFR mRNA expression **(A)** and pathway activation **(B)** of tumors harboring an EGFR amplification, activating mutation or wild-type. **(C)** Fraction of genetic alterations in tumors with an active *vs.* inactive EGFR pathway. Comparative PI3K mRNA expression **(D)** and pathway activation **(E)** of tumors harboring an PI3K amplification, activating mutation or wild-type. **(F)** Fraction of genetic alterations in tumors with an active *vs.* inactive PI3K pathway.

The multi-omic dataset of the TCGA-HNSC cohort enabled an integrative analysis of the impact of genetic alterations on EGFR and PI3K expression and pathway activity. EGFR amplified tumors showed a significantly higher expression (p < 2.22E-016, Student’s t-test), while this could not be observed for the few mutated cases (p = 0.700, Student’s t-test; **Figure 2A**). The difference in EGFR pathway activity between amplified and wild-type tumors was less pronounced (p = 0.017, Student’s t-test; **Figure 2B**), and the fraction of genetic alterations in the tumors with an active EGFR pathway was not significantly higher than the inactive group (15% *vs.* 10%, p = 0.141, Fisher’s Exact Test; **Figure 2C**). Conversely, the majority of HNSCCs (85%) with an activation of the EGFR signaling did not harbor an activating genetic alteration in EGFR. For PI3KCA, both amplifications and mutations resulted in an increased mRNA expression (p = 7.30E-12 and p = 0.016, Student’s t-test; **Figure 2D**). Interestingly, only mutations and not amplifications were found to result in a higher PI3K pathway activity (p = 0.0072 and p = 0.26, Student’s t-test; **Figure 2E**). The fraction of genetic alterations in PI3KCA was not significantly different between tumors with an active or inactive PI3K pathway (23% *vs.* 18%, p = 0.2369, Fisher’s Exact Test, **Figure 2F**). Consequently, 77% of HNSCCs with an activation of the PI3K signaling did not harbor an activating genetic alteration in PI3KCA.

### Identification of *In Vitro* EGFR and PI3K Pathway Models

**Figure 3:**
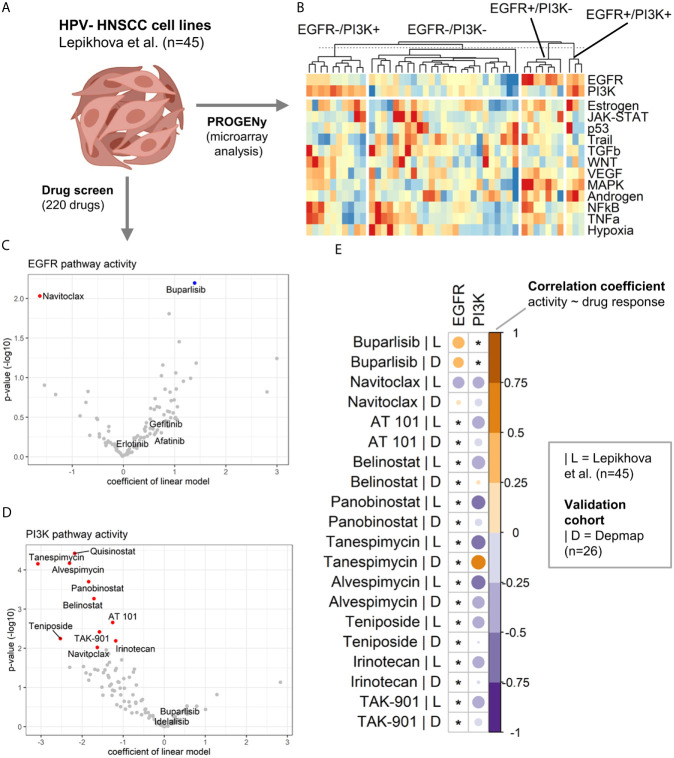
Drug sensitivity analysis in HNSCC cell line models of EGFR and PI3K pathway activation. **(A)** Transcriptomic and drug screen data of 45 HPV-HNSCC cell lines published by Lepikhova et al. was used. Created with BioRender.com. **(B)** Heatmap of pathway activity matrix of the HPV-HNSCC cell line cohort (n = 45). Samples were grouped by their EGFR and PI3K pathway activation status. **(C, D)** Volcano plots of the results from the linear modeling of drug responses for **(C)** EGFR and **(D)** PI3K pathway activity. A positive coefficient equals a positive association between pathway activity and drug sensitivity. For details regarding the modeling, please refer to the *Materials and Methods* section. Significant associations are colored in red (negative) and blue (positive). **(E)** Validation of the associations between pathway activities and drug responses in an independent validation cohort (DepMap data). The correlation heatmap compares the correlation coefficients of the initial cohort (|L = Lepikhova) and the validation cohort (|D = Depmap). An asterix marks associations that have not been significant in the initial cohort.

After having identified four pathway activity-based groups (EGFR+/PI3K+, EGFR+/PI3K-, EGFR-/PI3K+, and EGFR-/PI3K-) in HPV- tumors within the TCGA-HNSC cohort, it was investigated if suitable *in vitro* pathway models could be identified. To this end, the transcriptomic profiles of 45 HPV- HNSCC cell lines published by Lepikhova and colleagues (26) were analyzed (**Figure 3A**). Performing PROGENy on the normalized microarray data revealed pathway models for all four groups (**Figure 3B**).

### Drug Sensitivity Analysis

Lepikhova et al. performed a comprehensive drug screen (220 compounds) on the 45 HPV- HNSCC cell lines for which a gene expression microarrray analysis was available. As a measure of efficacy, the authors calculated a drug-sensitivity score (DSS). A higher DSS equals a higher efficacy at lower drug concentrations. The impact of EGFR and PI3K pathway activity on the drug response was modeled as a continuous parameter. Intriguingly, activation of the EGFR signaling did not predict for the EGFR inhibitors afatinib, erlotinib, or gefitinib (**Figure 3C** and **Table 1**). A high pathway activity was however associated with response to the PI3K inhibitor buparlisib (p = 6.36E-03). In contrast, a low pathway activity was predicted for response to the BCL inhibitor navitoclax (p = 9.26E-03). As for PI3K pathway activation, no tested compound was positively associated with drug response. An inactive PI3K pathway was associated with drug sensitivity in 10 drugs (**Figure 3D** and **Table 1**). The drug classes included two BCL, three HDAC, two HSP, two topoisomerase, and one aurora kinase inhibitor. In addition to individual EGFR and PI3K pathway activities, they were also modeled as a ratio (EGFR pathway activity/PI3K pathway activity). Here, a positive ratio (higher EGFR pathway activity) was associated with the response to buparlisib (p = 3.39E-04) and mitoxantrone (p = 6.83E-03; **Figure S6**). The statistics for all drugs are presented in **Table S3**.

**Table 1:** Cancer drugs significantly associated with EGFR and/or PI3K pathway activity.

**Name**	**Mode of action**	**Pathway association**	**P-value**	**Average DSS**
Buparlisib	PI3K inhibitor	EGFR+	6.36E-03	9.51
Navitoclax	BCL inhibitor	EGFR- | PI3K-	9.26E-03/9.44E-03	3.06
AT 101	BCL inhibitor	PI3K-	2.19E-03	12.66
Quisinostat	HDAC inhibitor	PI3K-	3.78E-05	17.29
Belinostat	HDAC inhibitor	PI3K-	5.40E-04	13.38
Panobinostat	HDAC inhibitor	PI3K-	1.99E-04	17.35
Tanespimycin	HSP inhibitor	PI3K-	6.978E-05	21.96
Alvespimycin	HSP inhibitor	PI3K-	6.72E-05	15.13
Teniposide	Topoisomerase inhibitor	PI3K-	5.62E-03	11.43
Irinotecan	Topoisomerase inhibitor	PI3K-	6.43E-03	3.11
TAK-901	Aurora kinase inhibitor	PI3K-	3.81E-03	8.93

The pathway association denotes the positive (+) or negative (-) association between the pathway activation and the drug efficacy. DSS, drug sensitivity score.

### Validation of Drug Response Associations in an Independent Cohort

HNSCC cell lines of the DepMap project were used as an independent validation cohort. As the HPV status for the 26 HNSCC cell lines is unknown, a published HPV gene expression signature (30) was investigated in the cohort indicating no HPV-positive cell line within the cohort (**Figure S7**). This analysis was complemented by a literature search that also did not identify a cell line as HPV-positive.

While the cohort size of the validation cohort was considerably smaller than the cohort by Lepikhova and colleagues, it allowed for an explanatory analysis. As a measure for the drug response associations between pathway activities of EGFR and PI3K and the drug response of the candidate drugs, correlation coefficients were calculated and compared (**Figure 3E** and **Table S4**). The drug screen data of the PRISM project included all candidate drugs with the exception of quisinostat. Based on this exploratory validation, the best concordance between the two studies could be observed for buparlisib|EGFR+ and alvespimycin|PI3K-. Less pronounced correlations but in the same direction could also be observed for navitoclax|PI3K-, AT 101|PI3K-, panobinostat|PI3K- and TAK-901|PI3K-. The only drug response association that was significantly in the opposite direction to the first cohort was tanespimycin|PI3K-.

## Discussion

EGFR is overexpressed in over 90% of head and neck tumors (31), and its expression is associated with a poor prognosis (32). Cetuximab, a monoclonal antibody targeting the extracellular domain of EGFR, remains a mainstay in the treatment of metastastic disease in combination with platinum and 5FU, but no predictive biomarker of efficacy has been developed. In contrast to the high fraction of protein overexpression, only 25% of samples in the TCGA-HNSC cohort were identified to have an active EGFR pathway. A recent phosphoproteomic study in HNSCC has identified that EGFR ligands rather than the receptor are the rate-limiting factor for EGFR pathway activity, offering an explanation for this discordance (33). This could be an explanation for the moderate efficacy of cetuximab in HNSCC (34, 35). Intriguingly, an active EGFR pathway did not predict for EGFR targeting tyrosine kinase inhibitors (afatinib, erlotinib, gefitinib), but instead for the PI3K inhibitor buparlisib. Buparlisib is the only PI3K inhibitor that has (in combination with paclitaxel) resulted in a clinical benefit in R/M HNSCC in the phase 2 BERIL-1 trial (12). EGFR alterations were not assessed in this trial. Buparlisib in combination with paclitaxel compared to paclitaxel alone is currently being assessed in the phase 3 BURAN trial for R/M HNSCC that have progressed after prior platinum-based therapy with or without prior anti-PD1/anti-PDL1 therapy (NCT04338399). An inactive EGFR pathway was identified to be associated with a response to the BCL inhibitor navitoclax. The BCL-2 family of proteins are key antiapoptotic factors that help tumor cells escape cell death (36) and could be shown to mediate resistance to tyrosine kinase inhibitors (37). Navitoclax is a BCL-XL/BCL-2 inhibitor and has already demonstrated efficacy in HNSCC cell lines (38, 39). However, it has been found to have limited *in vivo* activity in preclinical models of head and neck cancer (40).

PIK3CA mutations and amplifications are among the most common genetic alterations in HNSCC. While they occurred in more than 22% of all cases in the TCGA-HNSC cohort, 77% of HNSCCs with an activation of the PI3K signaling did not harbor an activating genetic alteration in PI3KCA. In the drug screen, PI3K activation was not found to be associated with a response to a PI3K inhibitor. This is in line with the results of the BERIL-1 trial that did not observe a higher response rate for buparlisib in tumors with a PI3KCA genetic alteration (41). The sensitivity of no tested drug was associated with a PI3K activation, but in turn a total of 10 drugs with an inactive pathway. The drugs included navitoclax that has also shown an association with an inactive EGFR pathway, as well as the BCL inhibitor AT 101. Another drug class were the histone deacetylase (HDAC) inhibitors quisinostat, belinostat, and panobinostat. HDAC inhibitors were shown to suppress the aggressiveness of HNSCC *in vitro* (42, 43). TAK-901 is an Aurora kinase inhibitor. Aurora kinases have recently been identified as therapeutic targets in EGFR-negative, gefitinib-resistant HNSCC cell lines (44). Intriguingly, Aurora kinase A (AURKA) has been shown to limit PI3K-pathway inhibition in a breast cancer model suggesting a dependent signaling network (45).

Published drug screens in HNSCC cell lines focused on the difference between HNSCC cell line and tumor cell lines derived from other entities (46), and the difference between HPV+ and HPV- cell lines (47) and investigated the impact of genetic alterations only (46). The drug screen of Lepikhova and colleagues (26) extended this view by analyzing HPV-negative cell lines and including gene expression analysis. The authors identified several associations between genetic alterations and drug responses, but did not make use of the expression data beyond the overexpression of individual genes. The disadvantage of genetic biomarkers is the intertumoral heterogeneity leading to a sparse biomarker information that impedes a drug prediction in every tumor (cell line). With our study, we took a transcriptome-based systems biology approach and successfully overcame this restraint.

However, a limitation of this study is the lack of a sufficiently large validation cohort for the drug-pathway associations. The cohort of Lepikhova presents one of the largest drug screens in transcriptionally defined HNSCC cell lines till date. A comparably large cohort of HNSCC cell lines (n = 42) is accessible *via* the Genomics of Drug Sensitivity in Cancer Project of the Sanger institute and the Massachusetts General Hospital (48). A validation of our key findings was however not possible as the main candidate drugs identified by our study were not part of the drug screen. We next aimed to validate our findings in the cell line cohort of the Cell Line Encyclopedia (CCLE) (28) but matching RNA-seq and drug screen data of the PRISM project (29) was only available in 26 HNSCC cell line samples. An exploratory correlative analysis revealed the best confirmation for the associations buparlasib|EGFR+ and alvespimycin|PI3K-. These findings require further validation in preclinical models and clinical studies.

## Data Availability Statement

The original contributions presented in the study are included in the article/**Supplementary Material**. Further inquiries can be directed to the corresponding author.

## Author Contributions

Conceptualization, AM, JH, and KZ. Methodology, AM, JH, and KZ. Formal analysis, AM and MT. Resources, SF, DJ. Data curation, AM, MP, and JM. Writing–original draft preparation, AM, MP, JM, JH, MT, and KZ. Writing–review and editing, DJ, JK, and SF. Visualization, AM and MT. All authors contributed to the article and approved the submitted version.

## Funding

AM is supported by the Physician-Scientist Program of the University of Heidelberg, Faculty of Medicine and is a fellow of the DKTK (German Cancer Consortium) School of Oncology and the Cancer Core Europe TRYTRAC program.

## Conflict of Interest

The authors declare that the research was conducted in the absence of any commercial or financial relationships that could be construed as a potential conflict of interest.
