# Supplementary material for: EGFR and PI3K Pathway Activities Might Guide Drug Repurposing in HPV-Negative Head and Neck Cancers
Source: Front Oncol. 2021 Jun 11;11:678966. doi: 10.3389/fonc.2021.678966 (PMC8226088; doi:10.3389/fonc.2021.678966)
Supplement: Supplementary file 1 [file DataSheet_1.docx]

SUPPLEMENTAL FIGURES

*EGFR and PI3K pathway activities might guide drug repurposing in HPV-negative head and neck cancers*

Mock et al.


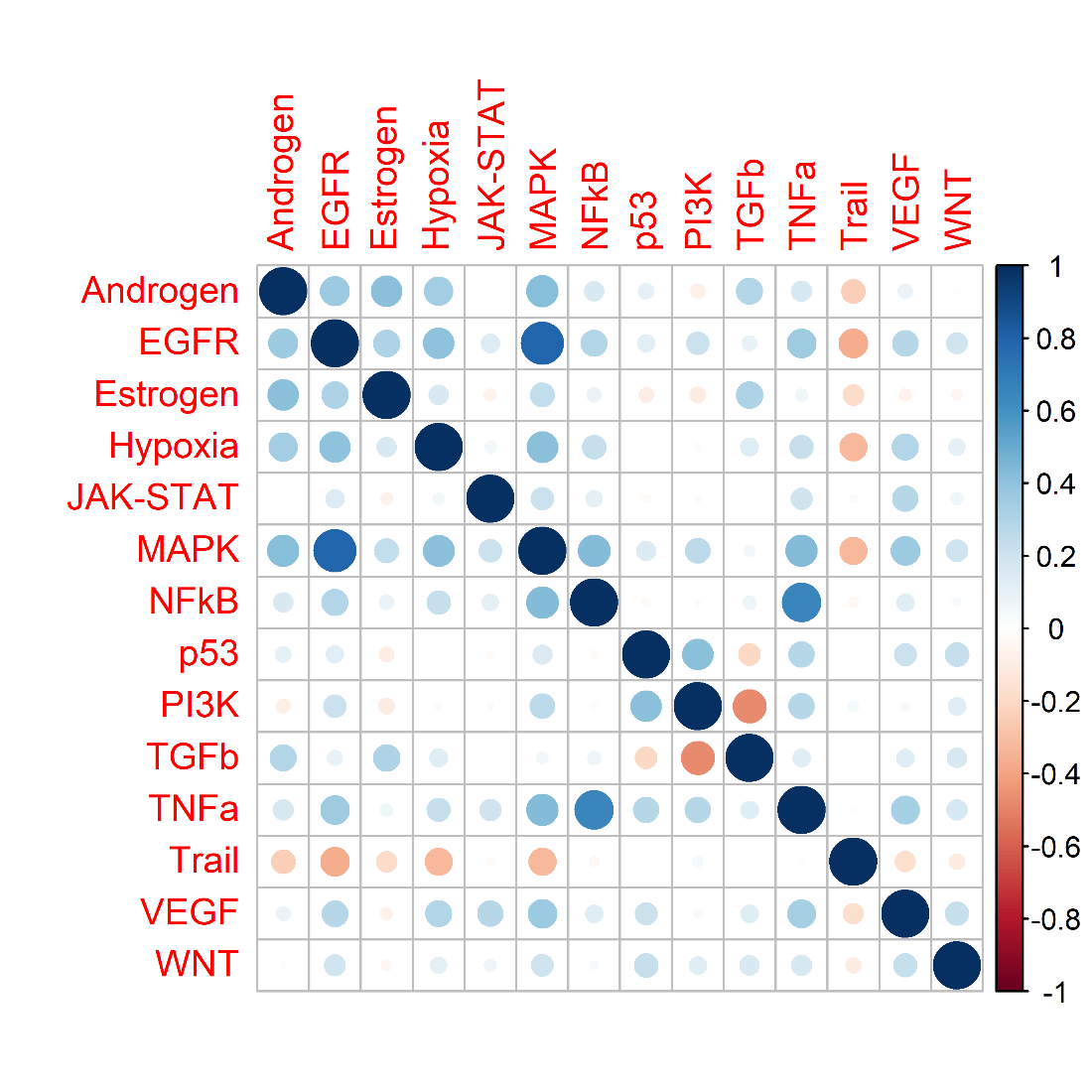


**Figure S1.** Correlation heatmap of inferred pathway activities in TCGA-HNSC cohort. The Pearson correlation coefficient is color coded (-1 red; 1 blue).


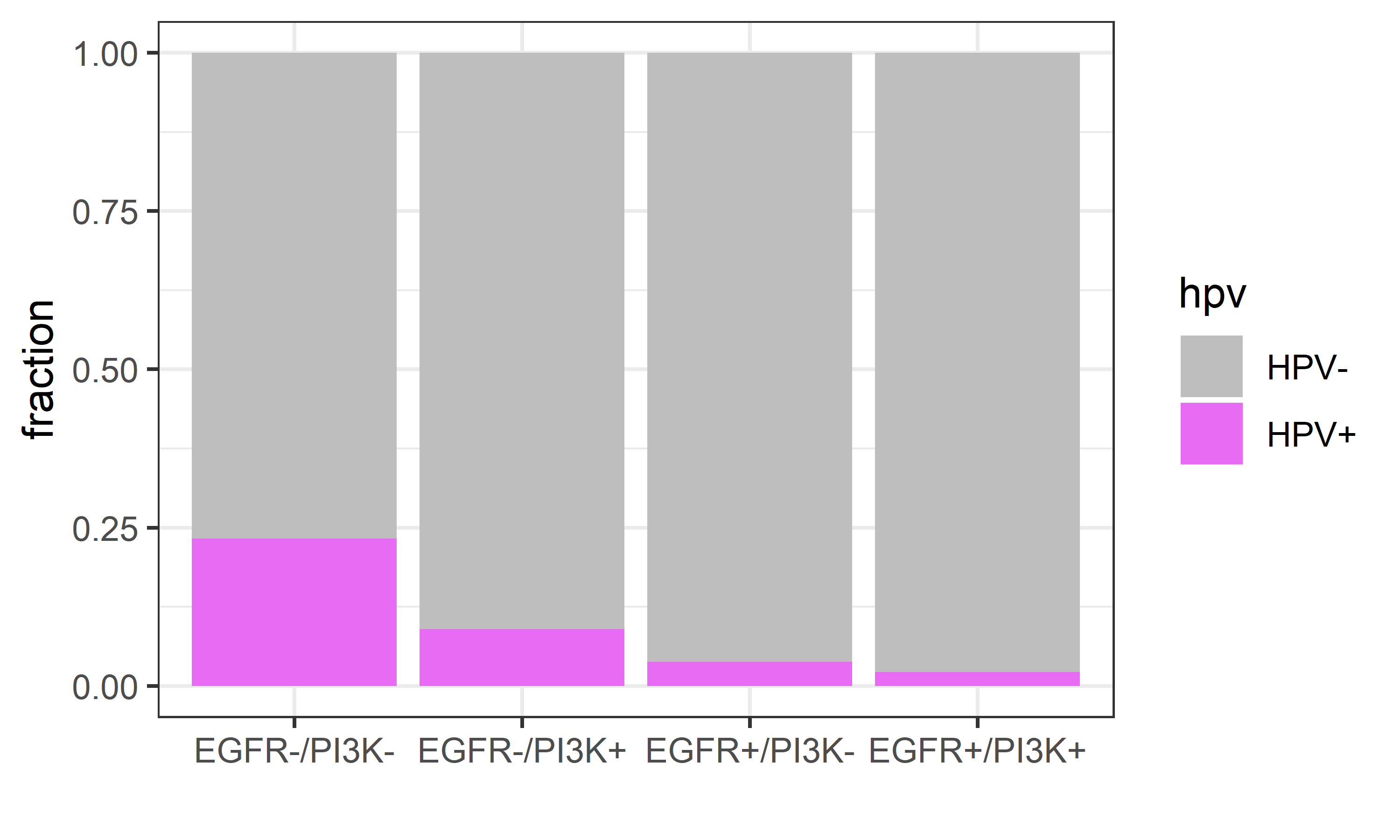


**Figure S2.** Fraction of HPV-positive cases in the TCGA-HNSC cohort across the four groups defined by pathway activation in EGFR and PI3K.


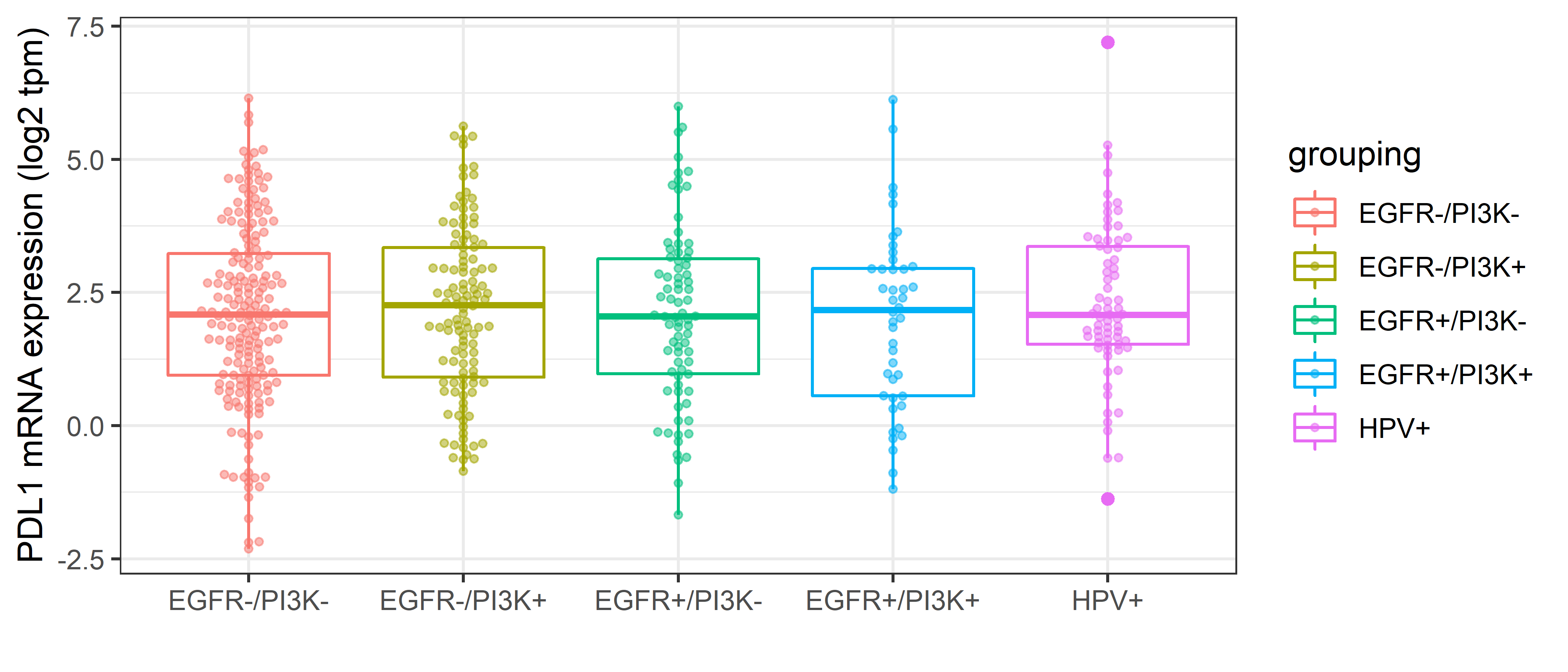
**Figure S3.** mRNA expression of PDL1 in the different subgroups in the TCGA-HNSC cohort.


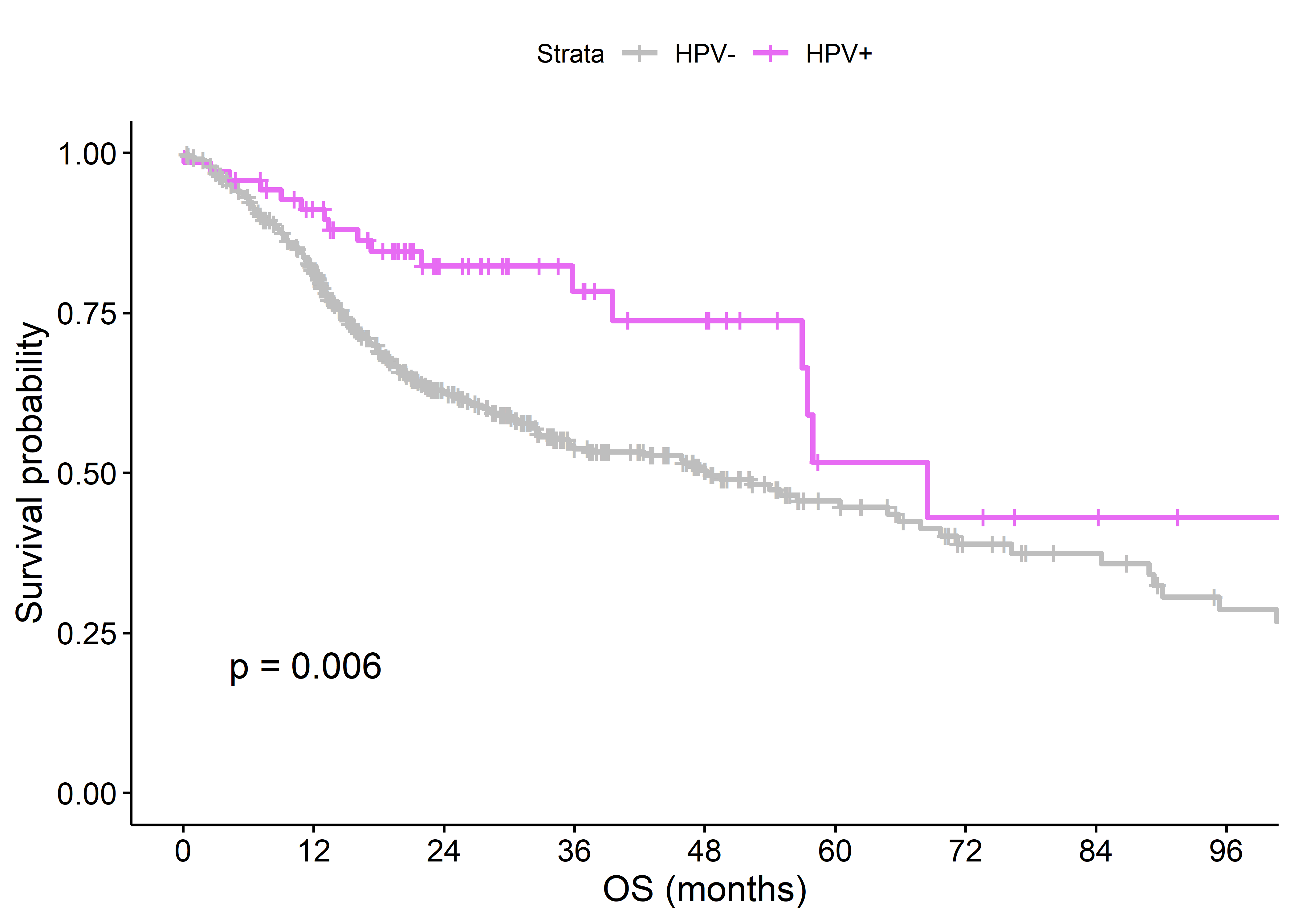

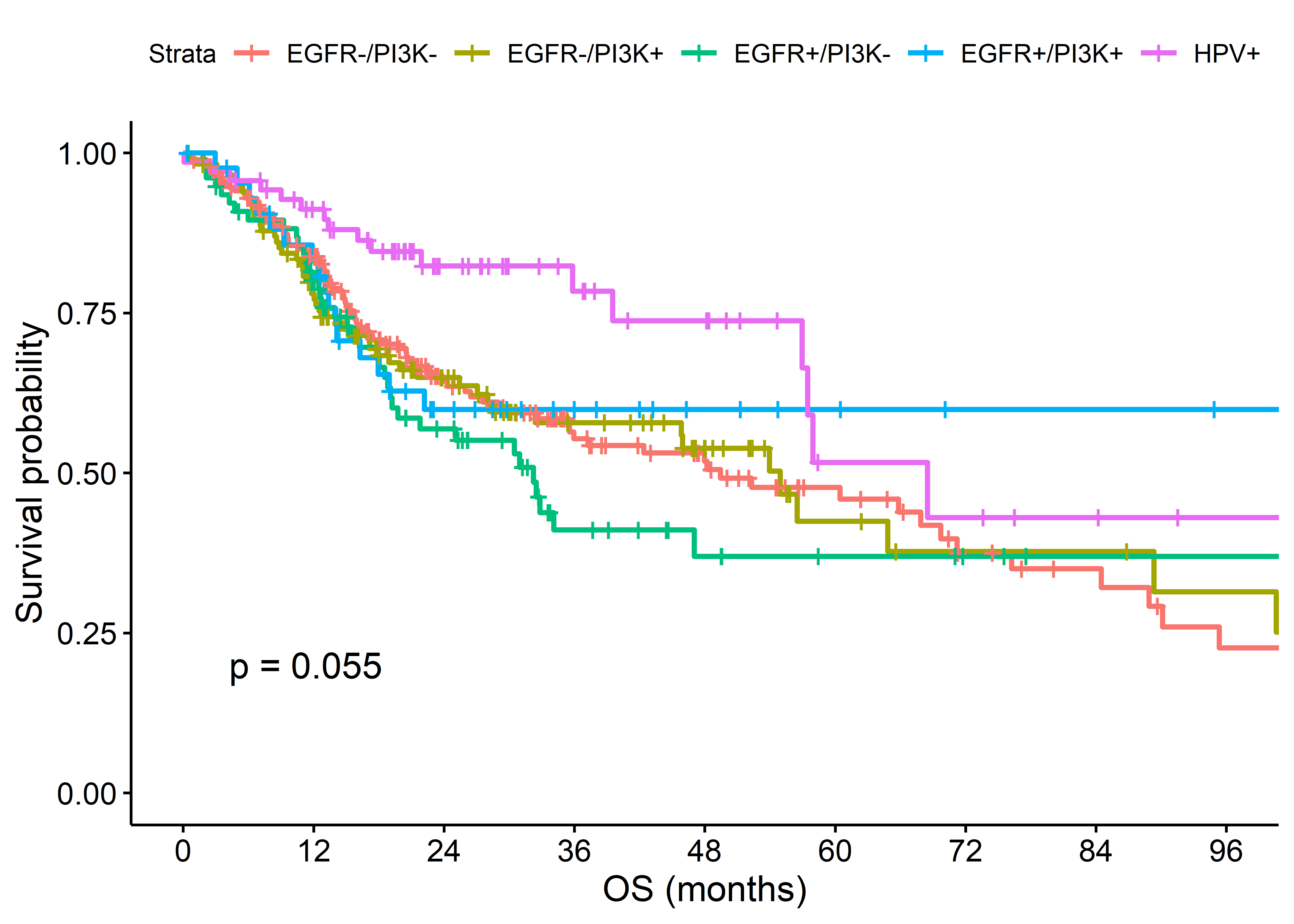


**a**

**b**

**Figure S4.** Kaplan-curves of the TCGA-HNSC cohort stratified by (a) pathway activation-based subgroups and HPV+ tumors and (b) by HPV status. P-values were calculated by log-rank test.


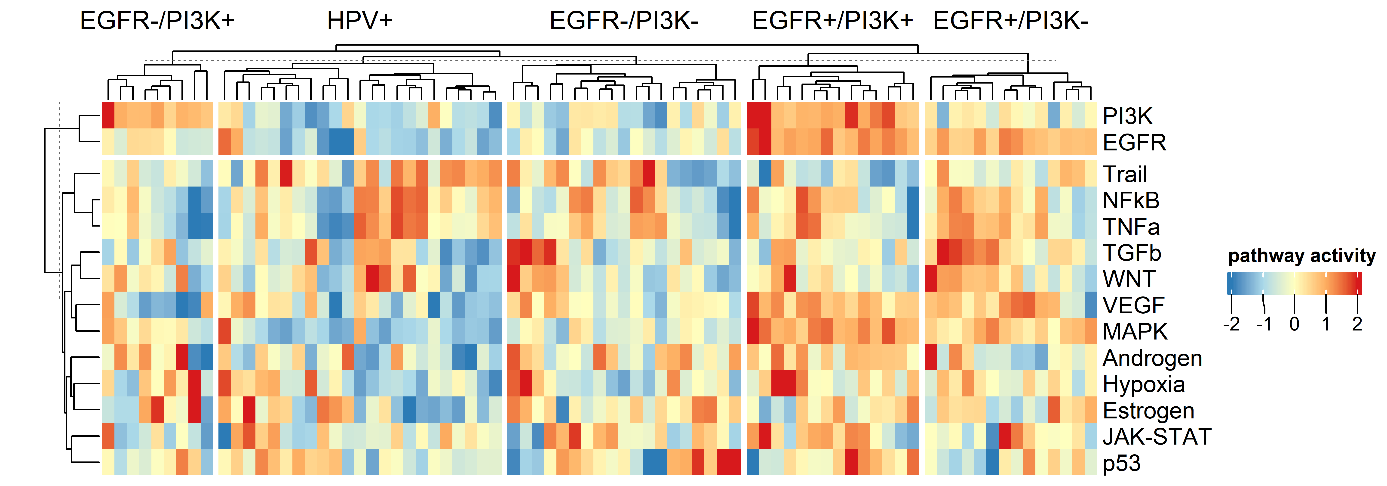


**Figure S5.** Heatmap of pathway activity matrix of the HIPO-HNSC cohort (n=79). Input to PROGENy were the -quantile normalized values of the gene expression microarray. Pathway scores were scaled across the cohort and a cutoff of >0.5 considered an activation. HPV+ samples were grouped separately irrespective of their EGFR and PI3K pathway activation.


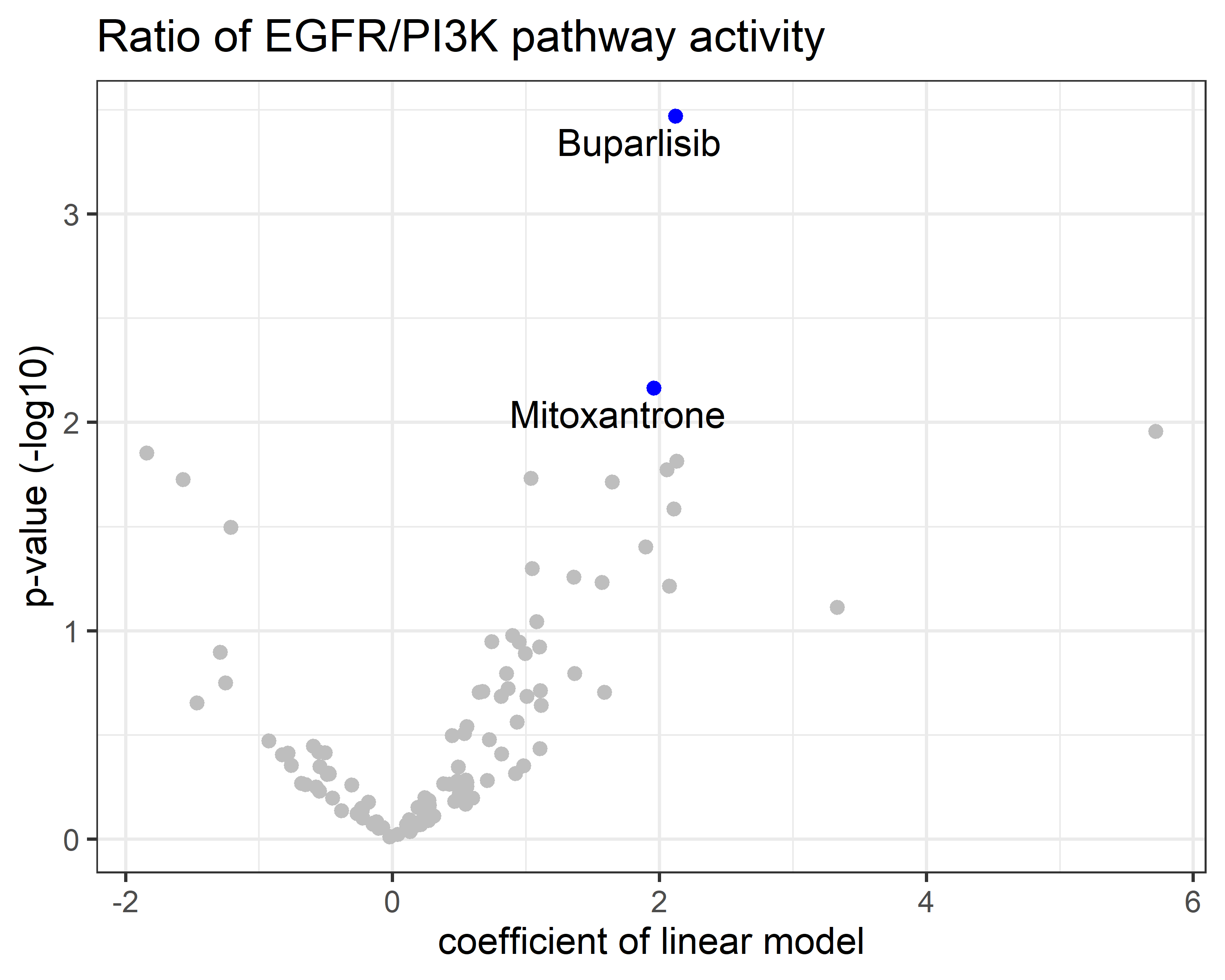


**Figure S6.** Volcano plot of the results from the linear modelling of drug responses for the ratio of EGFR and PI3K pathway activities (EGFR pathway activity / PI3K pathway activity).


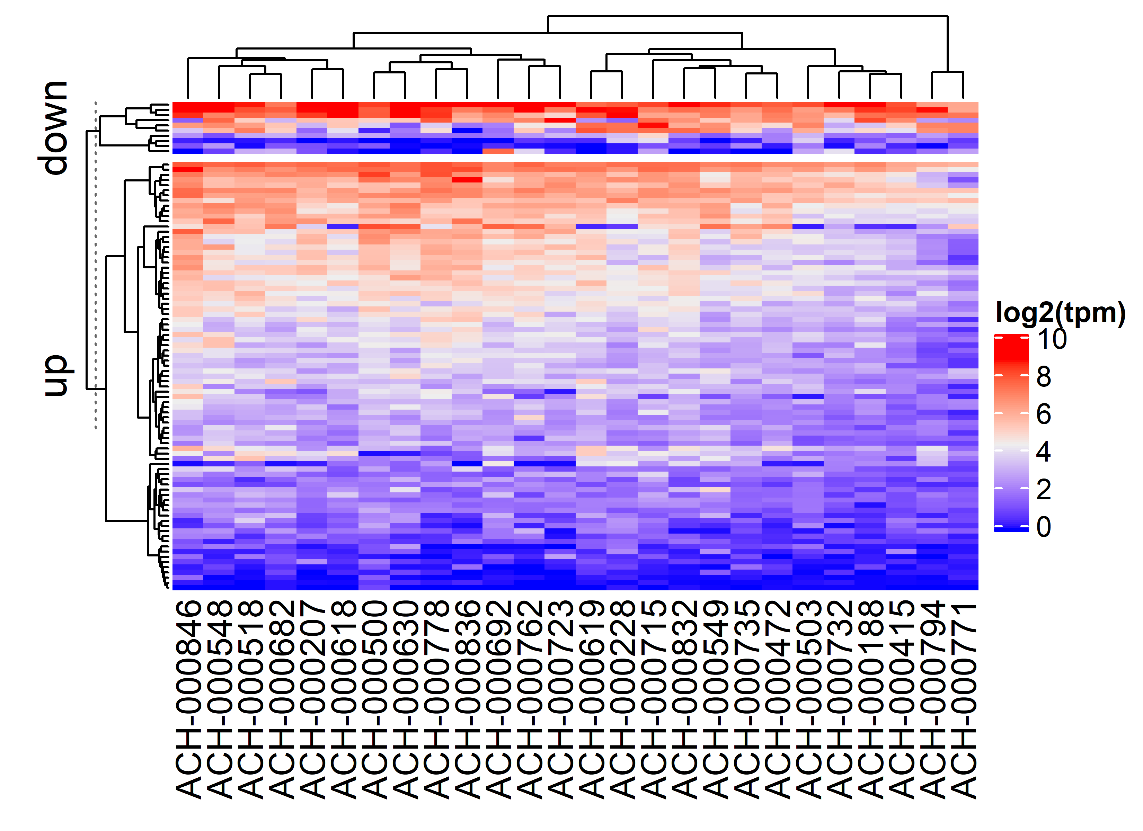


**Figure S7.** Heatmap of HPV signature genes identified by Pyeon et al.
